# No Free Lunch in Competency-Based Health Professions Education: Exploring Trade-Offs, Value, and Shared Investment in Health Professions Education

**DOI:** 10.5334/pme.2574

**Published:** 2026-09-09

**Authors:** Denise H. Rhoney, Michael A. Barone, Holly Caretta-Weyer, Laura Edgar, Daniel J. Schumacher, John Q. Young, Roberta Ladenheim, Kelly J. Caverzagie, For the International Competency-Based Health Professions Education Collaborators

**Affiliations:** 1 University of North Carolina Eshelman School of Pharmacy, Chapel Hill, North Carolina, USA; 2 American Board of Pediatrics, Chapel Hill, North Carolina, USA; 3 Stanford University School of Medicine, Palo Alto, California, USA; 4 Competencies, Milestones, and Faculty Development, Accreditation Council for Graduate Medical Education, Chicago, Illinois, USA; 5 Department of Pediatrics, Cincinnati Children’s Hospital Medical Center and University of Cincinnati College of Medicine, Cincinnati, Ohio, USA; 6 Northwell, New Hyde Park, New York, USA; 7 Department of Psychiatry, Donald and Barbara Zucker School of Medicine, Hofstra/Northwell, Hempstead, New York, USA; 8 Universidad Hospital Italiano de Buenos Aires, Juan D. Perón 4399, C.A.B.A, Argentina; 9 Oregon Health & Science University, Portland, Oregon, USA

## Abstract

**Background/Problem:**

Competency-Based Health Professions Education (CBHPE) aims to improve workforce readiness, patient safety, and equity, but achieving these outcomes requires sustained investment. The “No Free Lunch” (NFL) rule underscores that value cannot be realized without visible costs, which can be financial, cultural, temporal, and structural. Although costs are ideally shared across the health professions education and health care ecosystem, interest holders often invest unevenly despite broad benefits. As a result, many programs and institutions struggle to implement and sustain CBHPE, even as adoption grows globally.

**Purpose/Approach:**

This paper examines how “value” and return on investment (ROI) in CBHPE are conceptualized across learners, faculty, institutional leaders, governments/funders, clinical systems, accreditors/regulators, professional organizations, and patients/communities. The authors describe tensions regarding who invests and who benefits, identify practical trade-offs, and highlight opportunities for alignment across settings, including low-resource contexts. We propose an interest-holder-informed framework that links typical investments to forms of value/ROI to support explicit, context-sensitive cost-sharing.

**Key Insight:**

Value in CBHPE is contextual, shaped by the mission of the interest holder, the time horizon for returns, and available resources. Because benefits are often diffuse or delayed, ROI may be difficult to demonstrate, reinforcing underinvestment. Value must ultimately be judged by benefit to patients and communities.

**Conclusion:**

CBHPE cannot rely on the goodwill of those closest to implementation. Sustainability requires clear articulation of value, shared responsibility for costs that are proportional to benefits, and alignment between goals and resources among interest holders. CBHPE should be treated as long-term infrastructure for better care, not a temporary reform.

## Introduction

Despite decades of global effort, competency-based health professions education (CBHPE) has not fully achieved its intended goals of improving workforce readiness, enhancing patient safety, advancing equity among learners, and aligning training outputs with population health needs [1,2,3]. Persistent implementation gaps, including inadequate programmatic assessment systems, insufficient faculty development, entrenched time-based training structures, uneven regulatory expectations, and cultural resistance, continue to limit CBHPE’s ability to function as designed [4,5]. These barriers reflect not only technical challenges but also a deeper systems issue in which many interest holders assume that CBHPE can deliver transformative educational and societal outcomes without proportional investment in infrastructure, assessment, support, and organizational change [6,7].

A useful conceptual lens for understanding these shortcomings is the principle commonly known in North America as “No Free Lunch” (NFL), which holds that meaningful gains cannot occur without corresponding investments [8]. The expression originated from late-19th- and early-20th-century American saloons that advertised “free” meals only when customers purchased drinks, conveying the long-standing economic idea that every benefit carries an underlying financial, temporal, operational, or cultural cost. While the term “No Free Lunch” is North American in origin, the underlying principle- that meaningful benefits inevitably carry financial, temporal, cultural, structural, or political costs- is universal across health professions education systems. In many settings, these costs are most visible not as new line items but as trade-offs absorbed through workload, opportunity costs, and fragile infrastructure. Applied to CBHPE, the NFL principle highlights unavoidable trade-offs and opportunity costs, particularly when institutions attempt to implement competency-based systems in environments still structured around time-based progression and clinical service needs rather than learner development. Underinvestment, misaligned expectations, and insufficient shared responsibility among interest holders are recurring global barriers to effective CBHPE implementation, particularly across diverse geographic, economic, and regulatory contexts [9,10,11,12]. While the NFL principle emphasizes that meaningful gains require investment, this assumption applies after low-value or potentially harmful practices have been identified and eliminated; in such cases, discontinuing ineffective or harmful activities may create opportunities for improved outcomes without additional investment.

This manuscript uses the NFL principle to examine the real costs, investments, and value propositions inherent in implementing, executing, and measuring CBHPE’s effectiveness. We analyze how value, conceptualized as the relationship between benefits and the investments required to realize them, varies across interest holder groups, educational levels, resource settings, and regulatory environments. We further explore the trade-offs that emerge when CBHPE is introduced into systems still structured around historical models, including misalignment between expectations and resource commitments; gaps in assessment infrastructure; and shifting roles for faculty, learners, and institutions. Through a cross-interest holder analysis of benefits, required investments, and potential returns on investment, this manuscript provides a roadmap for educators, leaders, policymakers, and funders, outlining how CBHPE must be deliberately and equitably supported through shared investment models, aligned resource strategies, and the transparent articulation of value if its promised outcomes are to be achieved. Figure 1 summarizes this NFL framing by showing how interest holder value is shaped by context and value lenses, and why sustainability requires shared investment. Table 1 complements this model by operationalizing interest holder-specific investments, benefits received, and potential returns on investment across groups. This paper should be read primarily as a conceptual perspective, not as an economic evaluation, cost-effectiveness analysis, or prescriptive return on investment (ROI) model. We use value and ROI language heuristically to make visible the incremental relationships among investments, benefits, trade-offs, and shared responsibility as interest holders transition from existing educational systems toward CBHPE.

**Figure 1:**
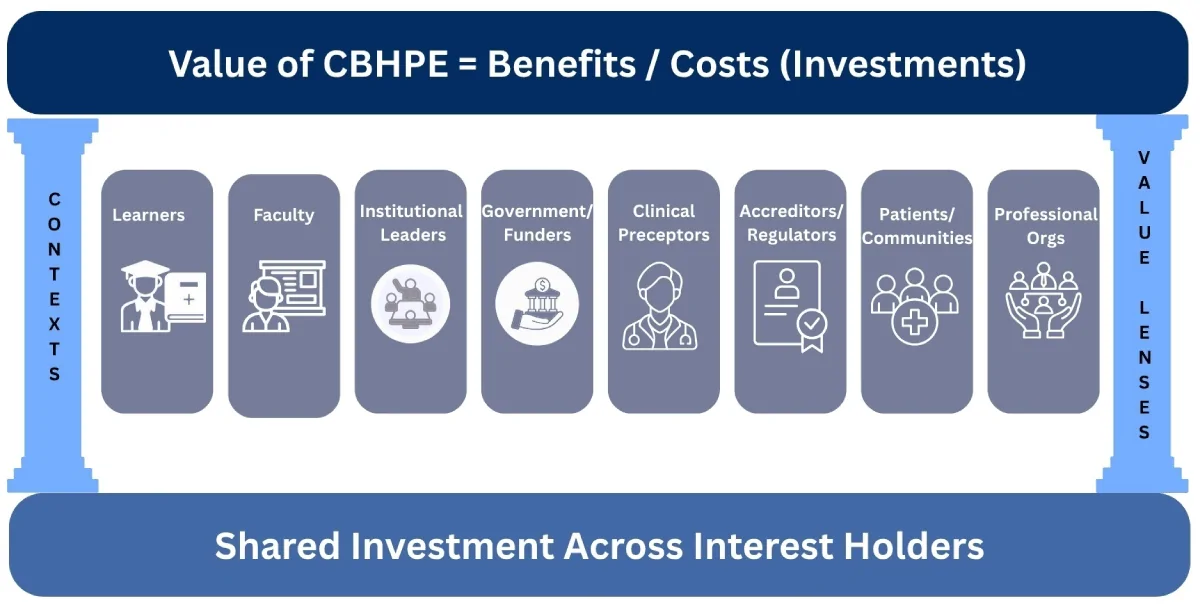
No Free Lunch in CBHPE: Interest Holder Value, Contexts, and Shared Investment. **Legend**: This conceptual model depicts how the value of competency-based health professions education (CBHPE) emerges from the relationship between benefits and costs (investments). The top bar states the value equation, Value of CBHPE = Benefits ÷ Costs (Investments). Eight interest holder groups (learners, faculty, institutional leaders, government/funders, clinical preceptors, accreditors/regulators, patients/communities, and professional organizations) are shown in the center. Two pillars support the interest holder row. The Contexts pillar highlights that local resources, technology, workload, and learner finances shape how benefits and costs are perceived. The Value Lenses pillar emphasizes that interest holders view value through economic, educational, cultural, and societal perspectives. The bottom bar, Shared Investment Across Interest Holders, underscores the No Free Lunch (NFL) principle: sustainable CBHPE requires aligning beneficiaries with who pays so that investments are distributed across interest holders rather than concentrated on programs and faculty.

**Table 1:** Interest Holder-Specific ROI Components in Competency-Based Health Professions Education (CBHPE).


INTEREST HOLDER	POTENTIAL ADDITIONAL INVESTMENT	POTENTIAL BENEFIT RECEIVED/RETURN FROM ADDITIONAL INVESTMENT

**Learners (Students/Residents)**	Tuition and fees and financial burden [14,15] Time and effort in self-directed learning [41,44,64] Engagement in feedback, coaching, and assessment processes [50,51,62] Consistent participation in program activities [52,53,64,66] Emotional labor, vulnerability in performance-based systems [43,45] Growth mindset (openness to feedback) [40,41] Adaptation to identity shifts and new expectations in CBHPE [20]	More personalized and developmentally appropriate learning pathways [1,2,30] Anticipated clearer expectations and progression toward competence [30] Improved readiness for practice and transition to responsibility [18,25,39,67] Potential for more efficient progression and earlier entry into practice roles [67] Reduced need for remediation and course repetition [62,63,64] Improved well-being and work/life balance [43,45] More frequent and meaningful feedback and coaching [52,60,66,81] Enhanced adaptive expertise for lifelong learning [40,41]

**Faculty (Academic & Clinical)**	Time for direct observation, feedback, and coaching [56,61,64] Participation in faculty development and calibration programs [59,60,82] Mentorship and longitudinal learner support [17,66] Engagement in curriculum design [54,55] Potential trade-offs with clinical productivity [19,68] Emotional and cognitive labor associated with coaching roles [62,81] Adapting to evolving roles and identity shifts required by CBHPE [20]	Improved teaching quality and more consistent assessment practices [58,62,63,81] Stronger relationships with learners and enhanced mentorship roles [66] Anticipated clearer alignment between teaching and learner outcomes [30,63] Enhanced career advancement, promotion, and recognition [25,59,68] Opportunities for scholarly work, mentorship, and educational leadership [17,19] Professional development and training aligned with CBHPE frameworks [60,66] Potential for improved patient care quality through supervised learner involvement [18] Increased professional satisfaction from meaningful educational roles [16,17,59,60]

**Program/Institutions (Educational Leaders)**	Investment in assessment systems and data infrastructure [19,52,53,73] Faculty development programs and protected faculty time [19,59,60,66] Hiring and retaining qualified educational and administrative staff [19] Strategic planning, policy development, and change management [10,11] Opportunity cost associated with reallocating resources from legacy systems [27,28]	Improved program quality, alignment, and accountability [18,38,39] Potential for stronger accreditation outcomes and regulatory compliance [10,11,30,83] Reduced remediation, delayed progression, and dropout rate [24,52,56,63,67] Potential for enhanced institutional reputation and prestige due to improved learner outcomes [1,2,6] More efficient and safer clinical learning environments [16,43,63,64] Anticipated improved patient care outcomes, including safety at the local level, leading to community trust [31,35] Long-term financial sustainability [19,27,28] Increased ability to attract high-quality faculty, staff, and learners [19,59]

**Governments & Funders**	Financial investment in education and training systems [27,28] Policy development and funding alignment [12,26] Investment in research, evaluation, and innovation initiatives [27,28] Strengthening accreditation, oversight, and quality assurance mechanisms [84,85] Opportunity cost associated with reallocating resources from legacy systems [19,27,28] Support for rural rotation programs, loan repayment, and targeted specialty funding [26,71,86,87]	Potential for improved population health outcomes [26,31,33,88] More efficient and reliable workforce pipeline [46,47,48,89,90] Measurable return on public investment [27,28] Better workforce alignment with societal needs (e.g., rural, primary care, shortages) [26,46,47,48,86,89,91] Increased public trust and accountability [21,84,85] Strengthened health system resilience and capability [46,47,48,88] Anticipated long-term health system sustainability and improved patient outcomes [31,32,33,88] Enhanced return on public investment in education and health care [27,28]

**Clinical Employers/Preceptors**	Time and resources for supervision, direct observation, and evaluation [52,63,64] Participation in workplace-based assessments and feedback cycle [62,81] Investment in onboarding and training programs [92] Shifting training to account for contextual variability within systems [18,80] Protected time for onboarding, coaching, and evaluation [58] Opportunity cost associated with reduced short-term productivity while supporting trainees [18,68]	More practice-ready graduates who require reduced onboarding and supervision [18,38,39,52,56,63,93,94] Increased workforce efficiency and reduced variability in performance [18,38,64] Improved team integration and communication [18] Improved patient safety and quality of care [35,52,63] Strengthened pipeline of future employees with increased retention and satisfaction [95]

**Accreditors/Regulators**	Development, refinement, and enforcement of competency frameworks [96,97] Adaptation of current accreditation and regulation standards to reflect CBHPE [96,97,98] Program evaluation and site visit [96,97] Data collection and analysis of learner performance and program data [52,53,96] Supporting continuous quality improvement initiatives [44,52,96] Investing in digital infrastructure for monitoring and reporting [52,53,96] Training reviewers and regulatory staff in CBHPE-aligned evaluation [96,97]	Opportunity for improved accountability and comparability of outcomes [30,96,103] Enhanced public confidence in educational and health systems [96,97] Fidelity to national/global frameworks [30,96] Clearer evidence for fidelity and accountability in training [52,96] More efficient quality assurance and regulatory oversight [96,97] Potential for earlier identification of competence concerns which may influence downstream remediation, disciplinary actions, or quality concerns. [44,52,96,97,98] Fewer unsafe practitioners entering the workforce [35,96]

**Patients & Communities**	Time and engagement in teaching and feedback roles [12,31,32,33] Engaging in community health initiatives [12,26] Advocacy for quality, accountability, and equity in local health systems [12,21,33] Participation in public forums or patient advisory councils [12,74] Sharing lived experiences to guide curricular and assessment design [12,74]	Improved safety, quality and equity of care [31,32,33,34,35,36] More patient-centered and culturally responsive care [31,33,34,35,36,37] Increased trust in healthcare systems and practitioners [21,84] Better alignment of care with community needs [12,31,33] Community influence on training priorities and communication skills [12,74] Empowered patients who are active partners in care improvement [33]

**Professional Organizations**	Advocacy and policy development at regional, national, or global levels [26] Creation of guidelines, best practices, and supportive resources [99] Support for continuing education, training, and innovation grants [85] Investment in research, best practices, and innovation [77] Developing and updating competency frameworks, entrustable professional activities (EPAs), and standards [30] Convening interest holders across disciplines for consensus building [100] Supporting registries, portfolios, and data systems for outcomes tracking [101]	Advancement of national and global standards of care [102] Strengthened professional identity and relevance [21] Enhanced reputation and public trust in the profession [84] Alignment with the evolving scope of practice and workforce needs across disciplines [18,46,47,48] Influence over policy, regulation, and professional norms [26] Increased member engagement, satisfaction, and retention [99] Ability to guide curricular standards, EPAs, and competency frameworks [30] Opportunity for expanded impact on patient outcomes through improved workforce readiness [104]


This table provides a conceptual mapping of potential interest holder-specific investments and the benefits or returns that may result from additional investment in CBHPE. Investments include financial, temporal, cultural/identity, and structural contributions. Benefits/returns include educational, clinical, workforce, societal, and equity-oriented value. The table is not intended as an economic model or quantitative ROI calculation; rather, it illustrates how additional investments may generate value across interest holder groups and why shared responsibility is needed. Note: The cited references reflect a combination of empirical evidence and literature describing proposed, expected, or theoretical benefits of CBHPE. As such, the table is intended to illustrate areas of demonstrated and potential value rather than to assert that all listed outcomes have been empirically established across contexts.

## The Value of CBHPE to Interest Holders: Context Matters

Value, in its simplest formulation, can be expressed as the relationship between what is gained and what must be invested to obtain that gain: Value = Benefit ÷ Cost [13]. We use this formulation as a conceptual organizing device rather than a quantitative equation, and we apply it primarily as an incremental value frame rather than a total or average value calculation. In other words, this manuscript focuses on the additional benefits gained and additional costs incurred when transitioning from existing, predominantly time-based educational models to CBHPE. This distinction is important because current systems already generate meaningful benefits and operate within established cost structures. In this manuscript, “cost” includes financial, temporal, cultural, structural, and opportunity costs, while “benefits” includes educational, clinical, workforce, societal, and equity-oriented value. Accordingly, the “worth” of adopting and maintaining CBHPE depends on how interest holders interpret what is newly gained, what must be newly invested or reallocated, and how those benefits and investments are distributed across the system.

Within CBHPE, value is not a singular or universal concept. Instead, it is a multidimensional construct shaped by interest holders priorities, institutional missions, regulatory pressures, and broader societal goals. Value may be conceptualized economically (e.g., cost of training vs. downstream health system savings), educationally (e.g., improved learner readiness and a developmental approach to achieve that goal), or societally (e.g., enhanced workforce alignment, improved population health outcomes, greater equity). Within interest-holder groups, value also varies contextually depending on the problem to be solved [13]. These varied interpretations highlight why a shared understanding of value is often difficult to achieve.

Because CBHPE touches the entire educational and clinical ecosystem, multiple interest holder groups must be considered when examining its value. These include learners, faculty, preceptors, administrators, accreditors, regulatory bodies, governments, funders, clinical employers, professional organizations, patients, and communities (Figure 1). Table 1 is intended as a conceptual mapping tool rather than an economic model. It illustrates examples of interest holder-specific investments, benefits received, and potential returns on investment to support explicit discussion of shared responsibility, while recognizing that actual costs, benefits, and funding mechanisms will vary by context. In fragmented education and health systems, common in many low- and middle-income countries, authority to invest, responsibility to implement, and benefit realization are often distributed across multiple interest holders. As a result, value may be widely shared while the ability (or willingness) to pay remains diffuse, creating predictable underinvestment even when interest holders conceptually support CBHPE.

Accordingly, interest holders hold legitimate yet distinct stakes in CBHPE and may define “value” differently based on their role, incentives, and constraints. For example, learners may view value through the lens of financial burden (e.g., loan debt in some settings; in others, the need to work during training, unpaid placements, and opportunity costs), time-to-practice, employability, flexibility, and well-being [14,15]. Faculty may emphasize work-life balance, teaching quality, and opportunities for mentorship and scholarly growth [16,17]. Clinical health system leaders may define value in terms of the services provided by learners and the financial and operational costs of training within systems operating under significant resource constraints. In some contexts, particularly in market-based systems, this may be experienced as narrow financial margins and rising costs relative to reimbursement rates. In publicly funded systems, similar pressures may be framed in terms of stewardship of limited resources, efficiency, and the need to demonstrate value and return on investment within constrained budgets [18]. Institutional program leaders may prioritize accreditation compliance, program sustainability, and reputation, while clinical employers may focus on workforce readiness, patient safety, and efficiency. Governments and funders may define value in terms of improved health outcomes, equity, and responsible stewardship of public resources. Regulatory bodies may emphasize accountability, achievement and comparability of outcomes, and protection of the public [19]. Professional organizations may foreground professional identity, public trust, and alignment with evolving scopes of practice. Patients and communities, the ultimate beneficiaries of health professions education, may define value in terms of competent, safe, equitable, accessible, and responsive care [20,21].

Importantly, context, including fragmentation of governance, funding responsibility, and benefit realization, profoundly shapes these perceptions of value. Interest holders in highly resourced environments may view value in terms of optimizing sophisticated assessment systems, simulation centers, faculty development programs, or digital learning platforms [11,19]. In contrast, interest holders in under-resourced settings may prioritize feasibility, affordability, minimal disruption to service delivery, and scalable solutions that do not rely on technology, subspecialty expertise, or large numbers of faculty [22,23]. Also, the perceived value of CBHPE may differ markedly between learners with financial security and those facing higher financial burdens, or between faculty with protected time and those balancing heavy clinical workloads [16,20]. Even within interest holder groups, perspectives may vary widely. For example, early learners prioritize flexibility and the speed of progression, while senior learners focus on transition-to-practice readiness [25]. In addition, perceived value may vary by local patient needs: rural communities with urgent access gaps may place greater emphasis on time-variable progression (a promise of CBHPE), whereas urban settings may place greater emphasis on ensuring learners have sufficient time and resources to develop competence before graduation [26]. Across contexts, underinvestment is rarely a “lack of belief” problem; it is more often a structural alignment problem, especially where fragmentation and hidden cost transfer make shared responsibility difficult to operationalize.

Collectively, these different perspectives, and likely many more, underscore that value in CBHPE is not static but rather a negotiated interpretation influenced by context, interest-holder needs, resource availability, and system constraints. As such, questions about who should invest in or “pay for” CBHPE implementation, maintenance, and improvement cannot be answered without understanding how the various interest holders perceive the benefits and costs. The NFL principle reminds us that no interest holder can claim value without proportional investment; benefits cannot be realized without costs borne somewhere, and by somebody, in the system [27,28]. In the sections that follow, we apply this cost-benefit lens to each interest-holder group and illustrate how value is distributed across settings, including examples from both low- and high-resource environments.

## The Benefits of CBHPE

The real and potential benefits of CBHPE are extensive. At its core, CBHPE is a patient-focused, learner-centered training model. Its central promise is to meet learners where they are, tailor instruction to individual developmental needs, and ensure that graduates are fully prepared to serve patients and communities. This individualized, outcomes-driven orientation forms the foundation for many of the benefits CBHPE aims to achieve [18,29,30]. The discussion below organizes these benefits into learner development and readiness for practice; downstream effects on care and system performance (Triple/Quadruple/Quintuple Aim); and program-level improvements enabled by programmatic assessment and continuous quality improvement.

Although individualized learner development is a defining feature, the advantages of CBHPE extend far beyond personalization. By translating explicit outcome competencies into sequenced progression, tailored learning experiences, competency-focused instruction, and programmatic assessment (CBHPE core components), CBHPE creates mechanisms that can reduce variability in practice readiness and strengthen feedback loops toward safer, more efficient, and more equitable care [29,30]. Accordingly, CBHPE aligns conceptually with the Triple (patient experience, population health, per-capita cost), Quadruple (clinician well-being), and Quintuple Aim (health equity) frameworks and may influence systems-level outcomes across patient care, clinician well-being, operational efficiency, and equity [31,32,33]. Consistent with the Quintuple Aim, CBHPE is also positioned to support an assessment paradigm oriented to safe, equitable, high-quality care, particularly when competency frameworks and programmatic assessment are designed to be culturally responsive and equity-informed [31,32,33,34,35].

Potential cost savings are often cited as a motivation for adopting CBHPE. By ensuring learners achieve competence in providing safe, efficient, and equitable care, CBHPE has the potential to reduce downstream costs associated with preventable errors, inefficiencies, and variable practice. There may also be cost implications for learners. CBHPE’s emphasis on time-variability, allowing learners to progress once competence is demonstrated rather than after a fixed duration, creates opportunities for financial benefit. Learners who progress more quickly may enter compensated phases of training or unsupervised practice sooner. Because time-variable pilots consistently show that early progression is more common than delayed progression, these financial gains may be meaningful for many learners and the training system as a whole [25,38,39].

Important benefits also arise from CBHPE’s learner-centered focus. First, CBHPE is designed to cultivate lifelong learning skills by helping learners develop the habits of self-assessment, reflection, feedback-seeking, and adaptive expertise. These capabilities strengthen future clinicians’ ability to remain current and responsive throughout their careers and may reduce significant gaps between evidence-based practice and current practice [40,41]. Second, CBHPE’s individualized approach acknowledges that optimizing competence should not come at the expense of learner well-being. A system built on continuous feedback, tailored support, and appropriate pacing has the potential to reduce stress, burnout, and hidden-curriculum pressures often associated with rigid, time-based training [42,43].

CBHPE’s emphasis on outcomes also yields curricular benefits. Modern CBHPE assessment systems frequently reveal patterns of underperformance or shared gaps across groups of learners. Identifying these recurring areas of struggle provides valuable information for programs, highlighting where curricula may be insufficient, misaligned, or ineffective, or where authentic learning opportunities are simply absent. This creates an evaluative feedback mechanism for programs, allowing educators to strengthen instructional design and assessment strategies in ways that more reliably achieve intended learning outcomes [44,45].

## Requisite Investments in CBHPE

The “No Free Lunch” principle highlights a fundamental truth of educational reform: although CBHPE promises better prepared graduates, stronger workforce alignment, and greater responsiveness to patient and population needs, realizing these benefits requires substantial investment. Importantly, many of the resources necessary for effective CBHPE implementation likely reflect the longstanding global underinvestment in health professions education, rather than a new or uniquely burdensome requirement introduced by CBHPE itself [46,47,48]. In this sense, CBHPE serves as a spotlight, exposing structural gaps, misaligned systems, and resource deficiencies that time-based models have long obscured or managed implicitly. Stated plainly, the investments we detail in this section should almost certainly already be the norm if HPE were funded to achieve even threshold goals [49,50,51].

Implementing high-quality CBHPE requires deliberate financial and human investment across infrastructure, curriculum, administration, and faculty preparation. Programs must establish systems capable of synthesizing longitudinal performance data, supporting individualized learning plans, and enabling programmatic assessment [52,53]. Although early commentary characterized these systems as inherently complex, contemporary implementation demonstrates that complexity often reflects institutional design choices (e.g., data architecture, assessment volume, committee workflows, delivery of feedback) rather than a defining feature of CBHPE. This distinction matters because it shifts the conversation from CBHPE is too complex to CBHPE must be intentionally engineered. Meaningful implementation also requires intentional curricular alignment, including mapping competencies, sequencing learning experiences, and removing redundant or outdated content. Curricular redesign is not an additive exercise layered atop existing structures but rather a necessary design that requires investment of time and system resources. Without streamlining legacy expectations, programs risk curricular congestion and learner and faculty fatigue [54,55].

Administrative demands also increase, as competency committees, progression decision-making, and accreditation documentation require structured governance and staff support [56,57]. These expectations are particularly challenging in low-resource contexts or in systems where clinical educators carry disproportionate administrative responsibility without dedicated time [58]. Faculty development introduces an additional category of investment [59]. High-quality direct observation, feedback, and coaching, which are central to CBHPE, are learned skills that require training, calibration, and ongoing support [60]. Opportunity costs must also be acknowledged, especially in clinical environments where faculty contribute directly to revenue generation; even small increments of teaching or assessment time represent meaningful trade-offs and thus a meaningful investment [61].

Beyond financial considerations, CBHPE introduces indirect and relational costs that shape interest holder experience. For learners, the transition from grades and fixed timelines to narrative assessment and the demonstration of competence may introduce considerable uncertainty [60]. However, evidence from systems with long-standing CBHPE practices, including the Netherlands, demonstrates that clear expectations, predictable assessment cycles, and consistent longitudinal coaching mitigate confusion and improve learner confidence. Individualized progression can also affect traditional cohort identity and peer comparisons, yet intentional community building, structured group learning, and developmental touchpoints can preserve a sense of belonging while accommodating flexible timelines [62,63].

Faculty often describe CBHPE assessment expectations as burdensome. However, empirical evidence shows that workplace-based assessments can be completed efficiently when tools are observationally anchored, high value, and integrated into workflows [64]. Perceived burden often stems from poorly designed, overly lengthy, cumbersome capture mechanisms or low-value assessment forms rather than from the core principles of CBHPE. The shift from faculty primarily delivering scheduled teaching sessions or supervising learners during clinical activities to faculty serving as coaches, observers, and longitudinal assessors of learner development also requires meaningful cultural and identity transitions. [65,66]. Without institutional recognition, protected time, and adequate support, which require investment, these changes may feel threatening or add strain. Institutional and cultural resistance may further complicate reform, particularly when CBHPE is layered onto largely unchanged time-based structures, thereby amplifying perceived burden and complexity without clear benefit [67,68].

Finally, CBHPE makes explicit the longstanding tension between clinical productivity and education. Time-based systems allowed this tension to remain implicit; CBHPE surfaces it directly. Institutions must determine how to value educational work alongside clinical demands, as prioritizing service at the expense of teaching undermines the integrity of CBHPE and the outcomes it aims to achieve. Some institutions and departments have attempted to recognize these contributions by introducing Educational Value Units, although widespread adoption has lagged [68].

When investments in CBHPE are not made explicit, costs are often displaced rather than avoided. They reappear as uncompensated faculty assessment labor, increased administrative load, reliance on goodwill, and “workarounds” that keep systems functioning without durable infrastructure. This hidden transfer can normalize fragility, and it is a central mechanism by which underinvestment persists even in systems that publicly endorse CBHPE.

In summary, value cannot be realized without cost, whether that cost appears as direct financial investment in a system, as sacrificed clinical productivity, or as hidden and indirect costs borne quietly by programs, faculty, and learners, as is currently the case. Thus, a fundamental question arises: who should pay for the costs of CBHPE? Historically, these costs have been absorbed implicitly by programs and faculty through increased workload, unfunded administrative responsibilities, and the repurposing of existing infrastructure rather than through intentional institutional or governmental investment. This approach may be tolerated temporarily, but it is increasingly incompatible with current productivity and workforce pressures. Learners, too, have shouldered costs due to time, uncertainty, and inefficiencies in systems that were never designed for true competency-based progression. Institutions contribute indirectly, but rarely at the scale required for systems-level redesign, and governmental or professional organizational support has been inconsistent across both local and global contexts. Finally, the most important interest holder, our patients, experience unacceptable variation in health care quality.

These realities illustrate a misalignment; while all interest holders (i.e., patients, health systems, professions, educators, institutions, and society) benefit from high-quality CBHPE, the costs have not been shared proportionally. Instead, each group often expects another interest holder to absorb the burden of costs, not realizing that these costs are actually investments in broader systems (i.e., CBHPE) that can work together to meet patient and societal needs. This expectation gap of viewing CBHPE as a cost, as opposed to an investment, is a key reason why, despite global momentum toward CBHPE, many programs and jurisdictions struggle to move from conceptual support to full implementation. Competing priorities, limited resources, and unclear funding models consistently stall meaningful forward progress.

## Interest Holder Value Received and the Shared Investment in CBHPE

If all interest holders benefit from CBHPE, how should the associated costs be shared? Stated differently, if all interest holders benefit, how might those interest holders invest in CBHPE? What might that return on that investment look like? How might these investments and their return vary within and between contexts? In many under-resourced settings, “ROI” is not the primary language of decision-making; CBHPE competes with immediate service-delivery pressures and workforce survival priorities. In addition, political and policy instability can make medium-term educational planning difficult, further weakening incentives to invest in CBHPE as long-term infrastructure even when there is agreement about its value. Investment can be the purchase of inexpensive tablets or mobile phones and the creation of simple educational videos to better educate healthcare workers in low-resource settings and countries, or the creation of high-intensity simulation centers and digital learning management centers in high-resource contexts [69,70,71,72]. Investment can be time and engagement with patients and communities who learn to teach and provide feedback to HPE learners, demonstrating that patients and communities can help learners to adapt their approaches to patients of different cultures and ultimately provide better care [74,41]. Across these examples, investment costs vary widely, yet the benefits commonly include enhanced training, assessment, workforce preparation, and care delivery [69,70,71,72,73,74].

Table 1 provides examples illustrating potential answers to these questions and others, including potential investments in CBHPE, the benefits received, and the potential return on that investment for key interest holders. These examples are not intended to be exhaustive, but rather to illustrate a range of possibilities for how investment in CBHPE, and that potential ROI, can be considered from the perspective of various interest holder groups without prescribing a uniform set of expectations.

Additional key points regarding interest holder value and share investment include:

- Investment in CBHPE should lead to a meaningful and, when possible, measurable ROI
- The relationship between investment and return is not always linear. As in most complex systems, additional investment may eventually yield diminishing marginal returns, and interest holders may differ in the level or type of return needed to justify further investment. These thresholds are shaped by competing priorities, resource constraints, and alternative investment opportunities across health care, education, and society.
- No single interest holder should be responsible for investing in the success of CBHPE, just as no single interest holder will receive all the benefits.
- Interest holder groups are not homogenous and may vary widely within a particular group (e.g., institutions vary in financial resources, governments differ in priorities, learners differ in their ability to absorb costs, and employers operate within different operational pressures)
- The value generated by CBHPE is shared across the entire system rather than a single interest holder group; therefore, the costs and investments required to sustain CBHPE should also be shared.

In summary, because the benefits of CBHPE are shared across the system, the responsibility for sustaining it should also be shared. This does not mean every group contributes in the same way or to the same degree. Rather, a coordinated approach in which each group contributes in ways that reflect its capacity, role, and potential to benefit is foundational. The table also underscores the limitations of the current reality. Programs, learners, and faculty, often the least resourced actors, typically shoulder a disproportionate share of the expected investment, even though many of the downstream gains accrue to employers, institutions, governments, and patients. Moving forward, effective cost-sharing models must account for this variation and adapt to local or national context, depending on the level(s) at or across which costs are being shared. The point remains, however, that CBHPE creates value across the entire health-professional education ecosystem, and sustaining that value requires a shared commitment to investing in its success.

## Limitations and Opportunities for Next Steps

Although this manuscript advances a system-wide argument for recognizing, funding, and sustaining CBHPE through shared investment, several limitations warrant acknowledgment. First, CBHPE is not a single model, but an approach to education that can be implemented within diverse regulatory, cultural, economic, and resource contexts. The framework we propose, rooted in the NFL principle and illustrated through the table, necessarily simplifies that variability. Interest holder groups are not monolithic, and the nature of value, cost, and investment differs across low-, middle-, and high-resource settings; between academic and clinical environments; and even among individuals within the same interest holder category. As such, our table represents conceptual examples rather than prescriptive or universally applicable models. The framework is therefore best understood as a conceptual scaffold for identifying and discussing trade-offs, not as a validated instrument for calculating ROI or comparing economic value across programs. Future work should refine these categories using empirical data from specific regions, professions, and institutional contexts to demonstrate how variable cost-sharing models might prove successful in different implementation environments.

Second, the current evidence base for CBHPE is still evolving and heterogeneous across outcomes and contexts. Emerging studies suggest improvements in learner readiness, assessment quality, workforce alignment, and system responsiveness; however, longitudinal evidence linking CBHPE to downstream patient outcomes, health equity, and workforce stability remains limited [75,76,77]. Similarly, economic analyses of CBHPE, whether focused on return on investment, cost avoidance, or social impact, are not yet fully established. These gaps constrain the specificity of cost-sharing recommendations and highlight the need for robust, multi-year, comparative evaluation. Importantly, CBHPE should be understood as one enabling strategy within a complex system rather than a single solution to all healthcare challenges; its value and impact will likely depend on complementary changes in policy, practice environments, and workforce supports. At the same time, broader implementation across diverse contexts is essential to generate the longitudinal and economic evidence needed to refine these recommendations.

The literature cited and perspectives emphasized in this manuscript largely reflect North American contexts, which is consistent with the authors’ positionality and the paper’s conceptual aims. This framework is offered as globally relevant rather than globally representative. It is intended as an invitation for contextual adaptation, critique, and empirical testing across diverse settings, including low- and middle-income contexts.

Despite these limitations, the manuscript also identifies clear opportunities for the next phase of work. Advancing CBHPE will require coordinated research to develop interest holder-specific return-on-investment metrics, comparative studies of financing models, and longitudinal tracking of educational and workforce outcomes. Policy alignment is needed across accreditation, regulation, and funding mechanisms to ensure that educational structures support learner progression based on competence, rather than time. Implementation science offers tools for understanding not only whether CBHPE works but under what conditions, for whom, and at what cost, expanding the field beyond conceptual enthusiasm toward measurable, durable impact [78,79]. Realist models may aid us in gaining a deep understanding of the contextual variability not only of CBHPE implementation but also of the contextual costs, benefits, and value achieved for a variety of interest holders [80].

Moving forward, the question is, therefore, not whether CBHPE has value but how that value can be realized through intentional, shared investment. Programs cannot continue to absorb the majority of the cost while governments, employers, and society benefit from a more capable and responsive health workforce. Nor can faculty shoulder disproportionate burdens without appropriate recognition and support. Ensuring the sustainability and scalability of CBHPE will require financial and structural commitment from all interest holders, guided by transparent definitions of value and context-sensitive strategies for distributing cost.

By articulating an interest holder-informed value framework, mapping potential investments and returns, and identifying future priorities for research and policy, this manuscript guides to help the health professions move toward a more equitable and sustainable model of educational transformation. CBHPE offers meaningful promise, but its promise can only be fulfilled if the system openly grapples with the question at the heart of the NFL principle: Who should pay for the benefits we collectively receive?

## Conclusion

CBHPE promises a more prepared and adaptable workforce that meets patient and community needs, yet achieving this value requires significant investment and shared responsibility. CBHPE’s benefits span learners, faculty, institutions, regulators, and patients, creating safer, more equitable care and stronger alignment between training and societal needs. Sustaining these gains demands collective action financing, or “investment,” and systemic redesign to ensure that all parties contribute according to their capacity and the value they receive from society.
